# Identification of Novel Anthracycline Resistance Genes and Their Inhibitors

**DOI:** 10.3390/ph14101051

**Published:** 2021-10-16

**Authors:** Onat Kadioglu, Mohamed Elbadawi, Edmond Fleischer, Thomas Efferth

**Affiliations:** 1 Department of Pharmaceutical Biology, Institute of Pharmaceutical and Biomedical Sciences, Johannes Gutenberg University, Staudinger Weg 5, 55128 Mainz, Germany; kadioglu@uni-mainz.de (O.K.); melbadawi@uni-mainz.de (M.E.); 2 Fischer Organics GmbH, 55413 Weiler, Germany; edmond.fleischer@gmx.de

**Keywords:** cancer, chemotherapy, drug resistance, RNA sequencing, transfection

## Abstract

Differentially expressed genes have been previously identified by us in multidrug-resistant tumor cells mainly resistant to doxorubicin. In the present study, we exemplarily focused on some of these genes to investigate their causative relationship with drug resistance. *HMOX1*, *NEIL2*, and *PRKCA* were overexpressed by lentiviral-plasmid-based transfection of HEK293 cells. An in silico drug repurposing approach was applied using virtual screening and molecular docking of FDA-approved drugs to identify inhibitors of these new drug-resistant genes. Overexpression of the selected genes conferred resistance to doxorubicin and daunorubicin but not to vincristine, docetaxel, and cisplatin, indicating the involvement of these genes in resistance to anthracyclines but not to a broader MDR phenotype. Using virtual drug screening and molecular docking analyses, we identified FDA-approved compounds (conivaptan, bexarotene, and desloratadine) that were interacting with HMOX1 and PRKCA at even stronger binding affinities than 1-(adamantan-1-yl)-2-(1H-imidazol-1-yl)ethenone and ellagic acid as known inhibitors of HMOX1 and PRKCA, respectively. Conivaptan treatment increased doxorubicin sensitivity of both *HMOX1*- and *PRKCA*-transfected cell lines. Bexarotene treatment had a comparable doxorubicin-sensitizing effect in *HMOX1*-transfected cells and desloratadine in *PRKCA*-transfected cells. Novel drug resistance mechanisms independent of ABC transporters have been identified that contribute to anthracycline resistance in MDR cells.

## 1. Introduction

Drug resistance is frequently multifactorial in nature, but the full complexity of mechanisms and genetic alterations has been rarely addressed. Multidrug resistance (MDR) is linked with drug efflux pumps such as P-glycoprotein, but other mechanisms are also involved [1,2,3,4]. Drugs accumulate in cancer cells by various mechanisms, such as diffusion, drug transport, and endocytosis. Each of these mechanisms possesses physiological significance based on detailed uptake studies in drug-resistant mutants [5]. 

**Figure 1:**
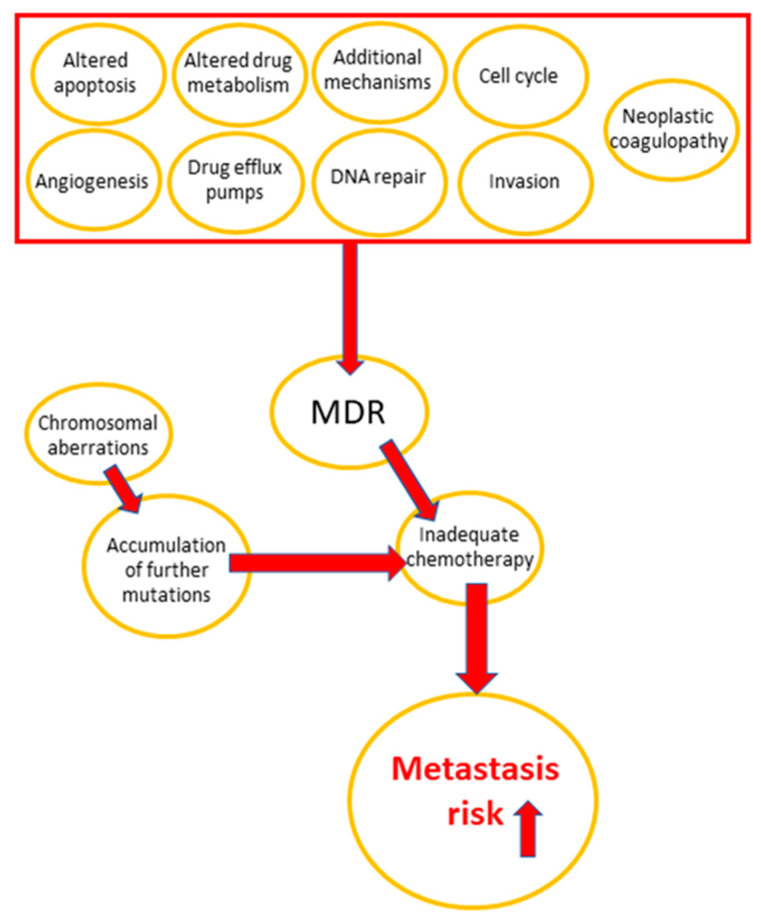
Effect of MDR-related mechanisms on cancer progression.

The main reasons of chemotherapy failure are drug resistance development in tumor cells and the high susceptibility of normal tissues to treatment-related toxicity [6,7,8,9,10]. Some important multidrug resistance mechanisms in cancer are apoptosis inhibition, DNA repair, drug efflux, and altered drug metabolism [5,11,12,13,14,15]. Vesicle trafficking, including the release of extracellular micro-vesicles, is critical in carcinogenesis and progression, which involves invasion, metastasis, cell cycle regulation, angiogenesis, tumor immune privilege, and chromosomal aberrations, all of which contribute to the development of multidrug resistance (MDR) [16]. The role of MDR mechanisms in cancer progression is visualized in Figure 1.

It has been reported that preventing or delaying the emergence of drug resistance potentially increases the effectiveness of chemotherapy and improves clinical outcomes for cancer patients [1]. In our previous study [2], we applied genomic and transcriptomic strategies to identify possible candidate mechanisms of drug resistance in addition to ABC transporters. To maximize the therapeutic benefit and minimize treatment-related toxicity, drug resistance phenomena should be better understood and the responsible mechanisms should be identified.

In the present study, we exemplarily selected candidate genes that were previously found to be overexpressed in multidrug-resistant CEM/ADR5000 tumor cells [2] in order to evaluate their causative relationship with MDR. A drug repurposing approach was applied to identify potential inhibitors of these novel drug resistance mechanisms in an attempt to overcome drug resistance conferred by these genes.

## 2. Results

### 2.1. Generation of Transfectant Cell Lines

**Figure 2:**
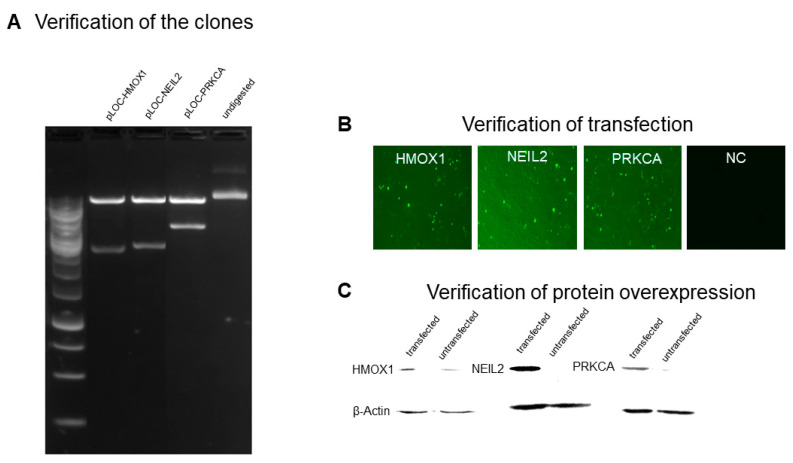
Transfection of HEK293 cells with *HMOX1, NEIL2*, and *PRKCA* plasmid constructs. (**A**) Verification of the clones after EcoRI digestion. (**B**) Verification of transfection with GFP signal observation under a fluorescent microscope. (**C**) Verification of protein overexpression for HMOX1, NEIL2, and PRKCA. β-actin was used as a loading control. NC, non-transfected control.

As shown in Figure 2, *HMOX1*, *NEIL2*, and *PRKCA* plasmid constructs were transfected into HEK293 cells. Expected bands were observed after EcoRI digestion of the plasmid constructs (Figure 2A). Successful transfection was verified with the GFP signal (Figure 2B). Overexpression of HMOX1, NEIL2, and PRKCA was verified for the transfected cells, and β-actin was used as a loading control (Figure 2C).

### 2.2. Resistance of Transfectant Cell Lines toward Anthracyclines

**Figure 3:**
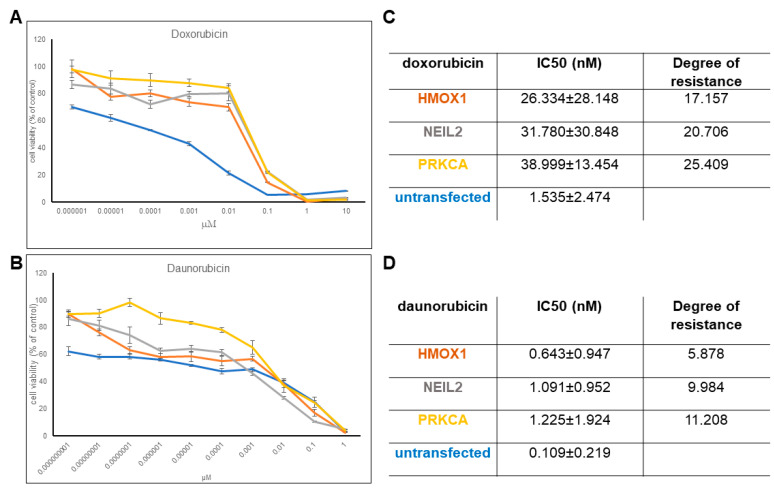
Effect of HMOX1, NEIL2, and PRKCA overexpression on doxorubicin and daunorubicin resistance. Dose response curves for (**A**) doxorubicin and (**B**) daunorubicinIC_50_ values for (**C**) doxorubicin and (**D**) daunorubicin treatment.

The established transfectant cell lines overexpressing either HMOX1, NEIL2, or PRKCA revealed increased resistance toward daunorubicin and doxorubicin (Figure 3), implying that the selected genes contributed to resistance to anthracyclines. We observed significantly higher IC_50_ values for *HMOX1*, *NEIL2*, and *PRKCA* transfected cell lines compared to the non-transfected cell line. It is clear from the corresponding dose response curves. The degree of resistance is especially higher for doxorubicin. Then, we concluded that upregulation of these genes leads to higher resistance toward anthracyclines, and we continued with analyzing the effect of potential HMOX1 and PRKCA inhibitors in the FDA-approved drug dataset to check whether they could induce a lower IC_50_ value for doxorubicin. The three transfected cell lines did not reveal resistance to vincristine, docetaxel, or cisplatin (data not shown).

### 2.3. Virtual Screening for HMOX1 and PRKCA Inhibitors

**Figure 4:**
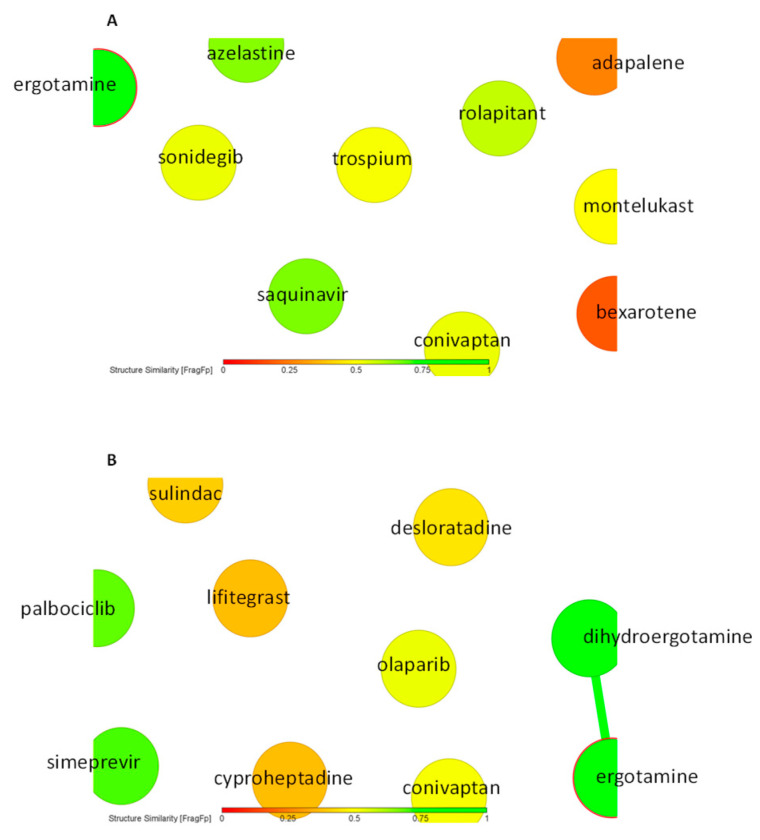
Similarity-based analysis of (**A**) top 10 FDA-approved drugs after HMOX1 screening and (**B**) top 10 FDA-approved drugs after PRKCA screening.

**Figure 5:**
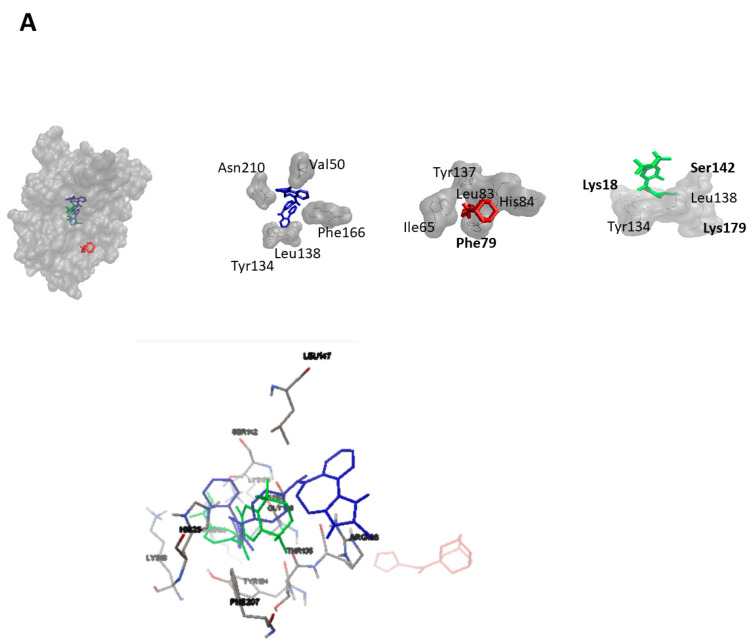

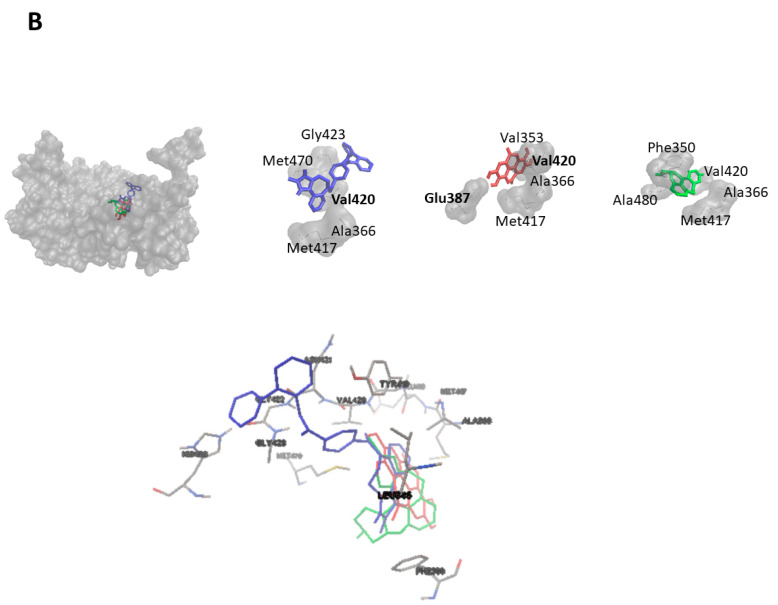
Molecular docking poses of conivaptan (blue) and bexarotene (green) on (**A**) HMOX1 and that of conivaptan (blue) and desloratadine (green) on (**B**) PRKCA. The known HMOX1 inhibitor 1-(adamantan-1-yl)-2-(1H-imidazol-1-yl)ethenone and the known PRKCA inhibitor ellagic acid are displayed in red. Amino acid residues forming hydrogen bonds are displayed in bold.

As a next step, we performed virtual screening with a library of 1577 FDA-approved drugs. The top 10 compounds with the highest binding affinity identified by PyRx-based blind docking using AutoDock VINA were selected and further subjected to defined molecular docking using AutoDock 4.2.6. The results for HMOX1 are summarized in Table 1 and for PRKCA in Table 2. His25, Leu147, and Phe207 are the most commonly observed residues and appeared for six compounds for HMOX1. Ala366 appeared for eight compounds, and Met417 appeared for seven compounds for PRKCA. The similarity analysis of the top 10 compounds with labels is depicted in Figure 4. Structural similarity analysis is based on the rubber banding forcefield approach, which translates similarity better than a principal component analysis (PCA) and is faster than a self-organizing map (SOM). It was performed to assess the similarities in the identified compounds. In this method, compounds are mapped into a two-dimensional area, similar molecules are located close to each other, and higher similarity is also indicated by green color. The structural diversity of the top 10 compounds from screening was reflected with the help of this similarity analysis. All test compounds revealed stronger binding affinities than the known control inhibitors. Conivaptan appeared in the top 10 list for both HMOX1 and PRKCA. The docking poses of conivaptan are visualized in Figure 5. Conivaptan and bexarotene bound to a slightly different region on HMOX1 compared to the inhibitor (1-(adamantan-1-yl)-2-(1H-imidazol-1-yl)ethenone), whereas conivaptan and desloratadine bound in close proximity on PRKCA compared to the inhibitor (ellagic acid).

There is no structure available for human NEIL2, and the sequence homology of human NEIL2 to the available NEIL2 structures from other species was low. Therefore, we did not perform homology modeling and subsequent in silico drug screening for NEIL2.

### 2.4. Doxorubicin-Sensitizing Effects of HMOX1 and PRKCA Inhibitors

**Figure 6:**
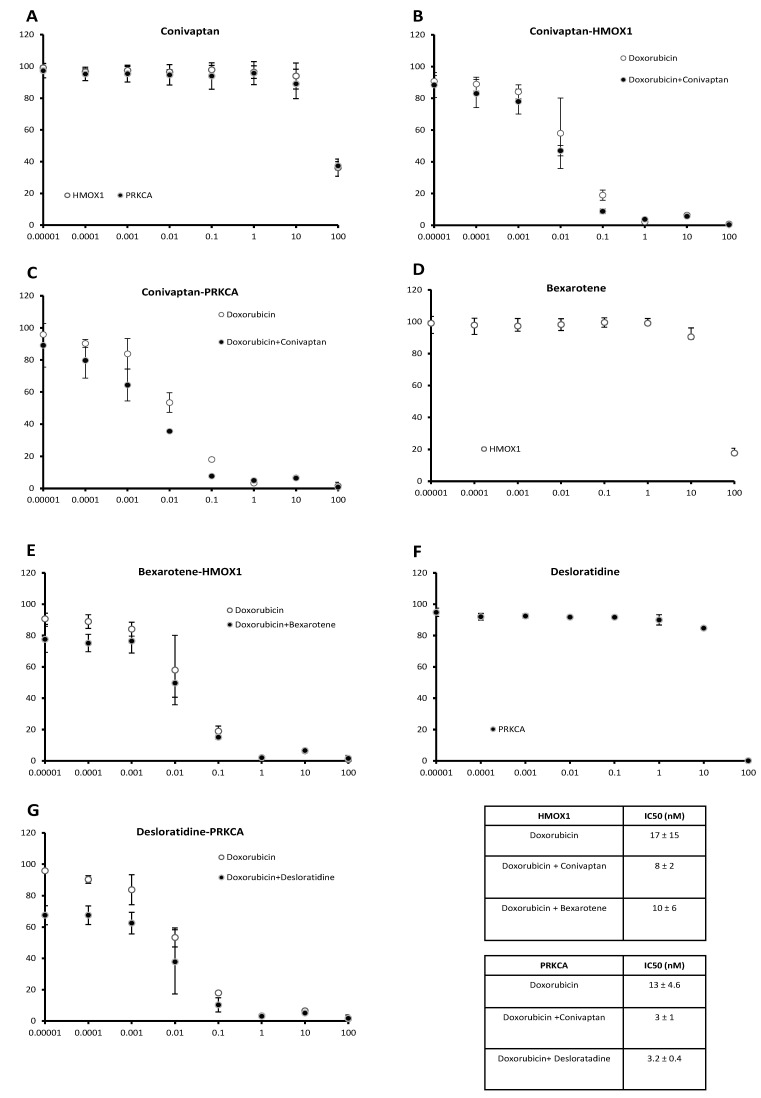
Effect of candidate inhibitors of HMOX1 or PRKCA on doxorubicin cytotoxicity in *HMOX1*- or *PRKCA*-transfected cell lines (cell viability, % of control: *y*-axis; concentration in µM: *x*-axis). (**A**) Conivaptan alone, (**B**) conivaptan with or without doxorubicin in *HMOX1*-transfected cells, (**C**) conivaptan with or without doxorubicin in *PRKCA*-transfected cells, (**D**) bexarotene alone, (**E**) bexarotene with or without doxorubicin in *HMOX1*-transfected cells, (**F**) desloratadine alone, and (**G**) desloratadine with or without doxorubicin in *PRKCA*-transfected cells.

Among the candidate compounds, conivaptan, bexarotene, and desloratadine caused clear decreases of IC_50_ values for doxorubicin in vitro, indicating sensitization of transfected cells toward doxorubicin by these three compounds. Treatment of both cell lines with conivaptan (10 µM) decreased the IC_50_ value for doxorubicin, implying an increased sensitivity toward doxorubicin. The dose response curves are shown in Figure 6. The IC_50_ values of conivaptan alone were 64.3 ± 10.6 µM for *HMOX1*-transfected cells and 60.4 ± 0.3 µM for *PRKCA*-transfected cells (Figure 6A). The IC_50_ values of conivaptan plus doxorubicin vs. doxorubicin alone in *HMOX1*-transfected cells were 8 ± 2 nM vs. 17 ± 15 nM (Figure 6B), and the IC_50_ values of conivaptan plus doxorubicin vs. doxorubicin alone in *PRKCA*-transfected cells were 3 ± 1 nM vs. 13 ± 4.6 nM (Figure 6C). Then, we treated the cell lines with bexarotene (10 µM). A doxorubicin-sensitizing effect was found on the *HMOX1*-transfected cell line. The dose response curve of bexarotene alone (Figure 6D) revealed an IC_50_ value of 35.8 ± 6.6 µM for *HMOX1*-transfected cells. Bexarotene plus doxorubicin vs. doxorubicin alone revealed IC_50_ values of 10 ± 6 nM vs. 17 ± 15 nM in the *HMOX1*-transfected cell line (Figure 6E). Desloratadine treatment (10 µM) revealed a doxorubicin-sensitizing effect on the *PRKCA*-transfected cell line. The dose response curve of desloratadine alone (Figure 6F) revealed an IC_50_ value of 27.5 ± 2.4 µM for *PRKCA*-transfected cells. Desloratadine plus doxorubicin revealed a lower IC_50_ value than doxorubicin alone in *PRKCA*-transfected cells (3.2 ± 0.4 nM vs. 13 ± 4.6 nM) (Figure 6G). 

## 3. Discussion

Drug resistance, in general, is a multifaceted phenomenon, and MDR is much more complex than frequently estimated. Beyond ABC transporters, there are more factors that account for the full mechanistical complexity of chemotherapy failure. For this reason, we previously performed transcriptomic and genomic profiling of multidrug-resistant tumor cells to investigate possible drug resistance mechanisms apart from ABC transporters in a comprising manner [2]. Among the upregulated genes, we selected some candidates to evaluate their causative involvement in drug resistance. 

One of these candidates was *HMOX1*. This gene encodes heme oxygenase, which metabolizes heme, CO, and ferrous iron. This enzyme regulates ferroptosis and is involved in tumor progression [17]. *HMOX1/HO-1* was 71.7-fold upregulated in CEM/ADR5000 leukemia cells [2] and has been linked with doxorubicin and daunorubicin resistance in the present study. HMOX1 has been previously shown to be involved in drug resistance of breast cancer cells by preventing apoptosis and autophagy, since siRNA knockdown of HMOX1 enhanced the cytotoxicity of doxorubicin in MDA-MB-231 and BT549 cells [18,19]. HMOX1 exerted anti-apoptotic activity in imatinib-resistant chronic myelogenous leukemia patients [20]. Inducing its expression via the PKC-β/p38-MAPK (mitogen-activated protein kinase) pathway promoted resistance of tumor cells to oxidative stress [20]. 

Another candidate gene chosen for the present study was *PRKCA*, which encodes protein kinase Cα. Protein kinase C members are involved in signal transduction pathways related to carcinogenic tumor promotion, cell adhesion, cell cycle control, etc. [21]. In the present study, PRKCA-transfected cells were resistant to anthracyclines unlike non-transfected cells. In our previous investigation of multidrug-resistant CEM/ADR5000 cells, PRKCA was 70.0-fold upregulated compared to CCRF-CEM sensitivecells [2]. PRKCA was also associated with drug resistance in ovarian cancer cells [22,23], colon cancer cells [24], and pancreatic cancer cells [25]. PRKCA phosphorylated and modulated the activity of RLIP76, which is involved in endocytosis of glutathione conjugates and xenobiotic compounds, including doxorubicin [26]. Inhibition of PRKCA and RLIP76 resulted in a synergistic increase of doxorubicin sensitivity [27].

A gene that was 22.3-fold upregulated in CEM/ADR5000 cells was *NEIL2* (Nei-like DNA glycosylase 2) [2]. NEIL2 is involved in DNA base excision repair by cleaving cytosine and other bases with oxidative damage [28,29]. DNA repair was also associated with the MDR phenotype of CEM/ADR5000 cells as 46 out of 225 DNA repair genes were deregulated, including *MSH4*, *BRCA2*, and *RRM2B*, in addition to *NEIL2* [2].

Previously, we characterized the cross-resistance profile of CEM/ADR5000 cells [10]. These cells were >1000-fold resistant to the resistance-selecting agent doxorubicin. CEM/ADR5000 cells were cross resistant to other typical drugs involved in the MDR phenotype (<500-fold), e.g., Vinca alkaloids and taxanes. While it seems plausible that the degree of resistance to doxorubicin as a selecting agent was higher than that to other cross-resistant drugs, the molecular mechanisms for this observation are unknown. Interestingly, three of the genes overexpressed in CEM/ADR5000 cells (*HMOX*, *NEIL2*, and *PRKCA*) conferred resistance to anthracyclines (doxorubicin and daunorubicin) but not to the other anticancer drugs tested if transfected into HEK293 cells. This indicates that the set of genes overexpressed in CEM/ADR5000 cells consist not only of genes that confer MDR to a broad spectrum of drugs, such as the ABC transporter P-glycoprotein, but also of genes that more specifically confer resistance to anthracyclines alone. This is an interesting and novel aspect of MDR cells that has been merely discussed in the past.

Our drug repurposing approach to identifying novel inhibitors for HMOX1 and PRKCA identified compounds with even stronger binding to these two targets than the corresponding known inhibitors of these proteins (1-(adamantan-1-yl)-2-(1H-imidazol-1-yl)ethenone and ellagic acid). Conivaptan was identified as an inhibitor of both HMOX1 and PRKCA among the top 10 out of the 1577 compounds investigated. Supporting the molecular docking results, conivaptan treatment indeed increased doxorubicin sensitivity of both transfected cell lines, implying its potential to be used as a dual inhibitor for both proteins. In addition, bexarotene sensitized HMOX1-transfected cells and desloratadine PRKCA-transfected cells to doxorubicin. Unfortunately, the unavailability of a suitable crystal or homology structure of NEIL2 prevented us from searching for NEIL2 inhibitors.

Conivaptan is a non-peptide inhibitor of vasopressin and was approved to treat hyponatriemia in neurological and neurosurgical patients as well as patients with heart failure [30,31]. Interestingly, it has also been used for hyponatriemia management in cancer patients and to prevent tumor lysis syndrome [32,33].

Bexarotene is a synthetic retinoid analogue with activity against cutaneous T-cell lymphoma and mycosis fungoides [34,35]. It has also been suggested to treat alopecia [36]. As alopecia is one of the most frequent side effects of cancer chemotherapy, this activity may be of interest if bexarotene would be considered for cancer therapy.

Desloratadine is an antihistaminic drug that suppresses allergic reactions, e.g., urticaria, rhinitis, and allergic inflammatory diseases [37,38]. Remarkably, desloratadine has been also reported to improve the survival time of patients suffering from melanoma or breast cancer [39].

Although all three drugs have been mentioned in the literature in the context of cancer, their relationship to anticancer drug resistance, as reported in the present paper for the first time, is novel. The sensitization of HMOX1- or PRKCA-overexpressing cells toward doxorubicin offers a thriving new option to improve the outcome of chemotherapy by overcoming anthracycline resistance. This study is of importance for the future development of more specific anticancer strategies for overcoming MDR.

## 4. Materials and Methods

### 4.1. Cell Culture

HEK293 cells were cultured in DMEM medium (Gibco, Eggenstein, Germany) supplemented with 10% FBS (Gibco), 1% penicillin/streptomycin (100 U/mL penicillin and 100 μg/mL streptomycin) (Gibco), and 6 mM L-glutamine (Gibco). 

### 4.2. Establishment of Stably Transfected Cell Lines

The main steps involved were as follows: (1) culturing of pLOC clones (Dharmacon, GE, Cambridge, UK) in ampicillin (Gibco) containing LB medium, (2) miniprep plasmid preparation with a QIAprep spin miniprep kit (Qiagen, Hilden, Germany), and (3) EcoRI (New England Biolabs, Frankfurt, Germany) digestion as described in the pLOC vector-based transfection manual mentioned in the manufacturer’s protocol. Three genes (HMOX1, NEIL2, and PRKCA) were selected from the list of upregulated genes in multidrug-resistant CEM/ADR5000 cells [2]. A pLOC lentiviral vector was used for the establishment of stably transfectant HEK293 cells. Transfected cells were selected by continuous blasticidin treatment (2.5 µg/mL, Gibco). Western blotting confirmed the overexpression of these three clones (HMOX1, NEIL2, and PRKCA). The protocol for Western blotting has been previously described [2]. Primary antibodies for HMOX1 and PRKCA (New England Biolabs, Frankfurt, Germany) as well as NEIL2 (Santa Cruz Biotechnology, Dallas, TX, USA) were diluted in a ratio of 1:1000. The primary antibody for β-actin (New England Biolabs) was diluted in a ratio of 1:2000. All antibodies were applied overnight at 4 °C. 

### 4.3. Cytotoxicity Assay

The resazurin assay [40] was applied to assess the cytotoxicity of established chemotherapy drugs toward the transfected cell lines. We performed the assay as recently described [41]. For each compound, three independent repetitions were performed with 72 h treatment. To investigate the sensitizing effect of in silico identified potential resistance modulating compounds, resazurin assays were performed by using non-cytotoxic concentrations of these compounds in combination with doxorubicin. Compounds presumably inhibiting HMOX1 or PRKCA were used to treat transfectant cell lines and study whether they were able to lower the IC_50_ values for doxorubicin.

### 4.4. In Silico Screening and Molecular Docking

A library of FDA-approved drugs (1577 compounds) was used to assess the binding strength toward HMOX1 (PDB ID: 1N45) and PRKCA (PDB ID: 3IW4), first by virtual screening with the AutoDock VINA algorithm. We converted the pdb files to pdbqt files to perform virtual screening and molecular docking. For the protein files, heterogenous molecules, such as water molecules, were removed to eliminate possible interference with the docking analysis. We performed virtual screening with AutoDock VINA, as it yields results faster, and we ranked the compounds according to their binding energies. Then we selected the best 10 compounds and performed molecular docking analysis to investigate the binding energies and docking poses with AutoDock by applying the Lamarckian genetic algorithm. The top 10 compounds with strong affinities were further considered for molecular docking in order to validate the binding energies and docking poses with the AutoDock 4.2 algorithm. As control drugs, 1-(adamantan-1-yl)-2-(1H-imidazol-1-yl)ethenone [42] was used as the known HMOX1 inhibitor and ellagic acid [43] was used as the known PRKCA inhibitor. Three independent repetitions were performed for both virtual screening and molecular docking.

### 4.5. Similarity Analysis

Data Warrior software (Actelion Pharmaceuticals Ltd., Allschwil, Switzerland, https://openmolecules.org/datawarrior/download.html, accessed on 22 September 2021) was used to perform the structural similarity analysis by referring to the user manual. Similarity Analysis available on this software uses a rubber banding forcefield approach, and it is stated to translate similarity better than a PCA and is faster than an SOM.

## 5. Conclusions

In conclusion, HMOX1, PRKCA, and NEIL2 contribute to anthracycline resistance within the more complex MDR phenotype, while P-glycoprotein/ABCB1 overexpression causes not only anthracycline resistance but also resistance to other anticancer drug classes [2]. Conivaptan has the potential to be used as the dual inhibitor of HMOX1 and PRKCA, whereas bexarotene has the potential as an HMOX1 inhibitor and desloratadine as a PRKCA inhibitor. All three compounds sensitized transfected cells to doxorubicin. 

## Figures and Tables

**Table 1:** In silico screening of FDA-approved drugs binding to HMOX1. Shown are the top 10 out of 1577 tested drugs. The amino acid residues in bold formed hydrogen bonds.

Compound	AutoDock 4.2.6 LBE (kcal/mol)	Interacting Amino Acid Residues
Adapalene	−12.383 ± 0.025	**Lys18**, His25, Ala28, Glu29, Thr135, Leu138, Gly139, **Ser142**, Leu147, **Lys179**, Arg183, Phe207, Asn210
Montelukast	−12.307 ± 0.671	**Lys18**, Thr21, Lys22, His25, Tyr134, Arg136, **Gly139**, Ser142, Gly143, Leu147, Lys179, **Arg183**, Phe207
Bexarotene	−11.527 ± 0.051	**Lys18**, His25, Tyr134, Thr135, Arg136, Leu138, Gly139, **Ser142**, Leu147, **Lys179**, Arg183, Phe207
Conivaptan	−10.837 ± 0.427	His25, Val50, Leu54, Ile57, Tyr134, Arg136, Leu138, Gly139, Asp140, Ser142, Gly143, Leu147, Phe166, Phe167, Phe207, Asn210, Leu213, Phe214
Sonidegib	−10.420 ± 0.226	**Val59**, Glu62, Glu63, Ile65, Glu66, Val77, Tyr78, Phe79, Pro80, Leu83, His84, Lys86, Tyr137
Trospium	−10.010 ± 0.044	His25, Met34, Phe37, Phe47, Val50, Leu54, Thr135, **Arg136**, **Asp140**, Leu147, Phe167, Phe207, Asn210
Azelastine	−9.950 ± <0.001	His25, Ala28, Glu29, Met34, Gln38, Val50, Thr135, **Arg136**, Leu147, Phe207, Asn210, Phe214
Ergotamine	−9.877 ± 0.035	Tyr55, His56, Val59, Tyr107, Gln112, Arg113, Val115, Lys116, His119
Saquinavir	−8.180 ± 0.370	Leu49, Tyr97, Gln102, Glu103, Val104, Ile105, Pro106, **Tyr107**, Thr108, Pro109, Gln112, Leu220
Rolapitant	−7.727 ± 0.156	Glu62, Glu63, Ile65, Glu66, Tyr78, Phe79, Pro80, Leu83, His84, Lys86, **Tyr137**
1-(Adamantan-1-yl)-2-(1H-imidazol-1-yl)ethenone	−7.333 ± 0.032	Glu62, Ile65, Glu66, Tyr78, **Phe79**, Pro80, Leu83, His84, Tyr137

**Table 2:** In silico screening of FDA-approved drugs binding to PRKCA. Shown are the top 10 out of 1577 tested drugs. The amino acid residues in bold formed hydrogen bonds.

Compounds	AutoDock 4.2.6 LBE (kcal/mol)	Interacting Amino Acid Residues
Lifitegrast	−13.080 ± 0.123	His455, Lys456, Met489, Asp491, Gly492, Tyr515, Gly516, Lys517, Ser518, Pro577, Gly587, Glu588
Conivaptan	−12.207 ± 0.015	Leu345, Phe350, Ala366, Met417, Glu418, Tyr419, **Val420**, Asn421, Gly422, Gly423, His428, Met470
Dihydroergotamine	−11.187 ± 0.061	Leu345, Phe350, Val353, Ala366, Lys368, **Glu387**, Met417, **Tyr419**, Val420, Asn421, Met470, Ala480, Asp481
Olaparib	−10.763 ± 0.042	Phe350, Val353, Ala366, Lys368, Glu387, Thr401, Met417, Tyr419, **Asp467**, Asn468, Met470, Ala480, **Asp481**
Simeprevir	−10.663 ± 0.086	Leu345, Val353, Ala366, Tyr419, Val420, **Asn421**, Gly422, Gly423, **Asp424**, Tyr427, His428, Met470, Lys617
Ergotamine	−10.663 ± 0.291	Leu393, Leu394, Asp395, Lys396, Pro397, Pro398, Gln402, **Leu403**, Ile449, Phe453, Glu606, Asn607
Desloratadine	−10.450 ± <0.001	Phe350, Ala366, Lys368, Met417, Tyr419, Val420, Asn468, Ala480, Asp481
Palbociclib	−9.810 ± 0.0529	Phe350, Ala366, Lys368, Glu387, Leu391, Thr401, Met417, Glu418, Val420, **Asp424**, Asp467, Met470, Ala480, Asp481
Cyproheptadine	−9.760 ± <0.001	Gly346, Phe350, Val353, Ala366, Lys368, Glu387, Thr401, Met417, Glu418, Val420, Asp481
Sulindac	−9.207 ± 0.183	**Lys347**, Gly351, **Lys352**, Ile369, Leu370, Lys371, Val374, Val375, Asp378
Ellagic acid	−7.550 ± <0.001	Val353, Ala366, Lys368, **Glu387**, Thr401, Met417, **Val420**

## Data Availability

Data is contained within the article.

## Abbreviations

ABC, ATP-binding cassette; FDA, Food and Drug Administration; HMOX1, heme oxygenase 1; MDR, multidrug resistance; NEIL2, Nei-like DNA glycosylase 2; PRKCA, protein kinase C α.
